# Statement of Retraction: F-box and WD repeat domain-containing 7 (FBXW7) mediates the hypoxia inducible factor-1α (HIF-1α)/vascular endothelial growth factor (VEGF) signaling pathway to affect hypoxic-ischemic brain damage in neonatal rats

**DOI:** 10.1080/21655979.2024.2299599

**Published:** 2024-02-20

We, the Editors and Publisher of the journal Bioengineered, have retracted the following article:

Ling Sun (2022) F-box and WD repeat domain-containing 7 (FBXW7) mediates the hypoxia inducible factor-1α (HIF-1α)/vascular endothelial growth factor (VEGF) signaling pathway to affect hypoxic-ischemic brain damage in neonatal rats, Bioengineered, 13:1, 560-572, DOI: 10.1080/21655979.2021.2011635

Since publication, significant concerns have been raised about the integrity of the data and reported results in the article.

When approached for an explanation, the author did not respond, nor did they provide their original data or any necessary supporting information. As verifying the validity of published work is core to the integrity of the scholarly record, we are therefore retracting the article. The author listed in this publication has been informed.

We have been informed in our decision-making by our editorial policies and the COPE guidelines.

The retracted article will remain online to maintain the scholarly record, but it will be digitally watermarked on each page as ‘Retracted’.

